# Preparation and Properties of AgNPs Loaded CMC-CMCS Composite Packaging Films with Dual Reduction and Stabilization Functions

**DOI:** 10.3390/polym18172174

**Published:** 2026-09-06

**Authors:** Chenrong Yang, Pan Xie, Hao Zhang, Xinbo Yu, Yongheng Qiao, Yuting Liu, Guiqiang Fei, Haihua Wang

**Affiliations:** 1 Shaanxi Key Laboratory of Chemical Additives for Industry, Shaanxi University of Science and Technology, Xi’an 710021, China; 2 Shandong Inov Polyurethane Co., Ltd., Zibo 255000, China; 3 College of Chemistry, Chemical Engineering and Materials Science, Soochow University, Suzhou 215123, China

**Keywords:** carboxymethyl chitosan, silver nanoparticles, ternary composite film, antibacterial activity, food packaging

## Abstract

Food packaging materials require both antibacterial activity and mechanical strength to ensure food safety. However, conventional synthesis of silver nanoparticles (AgNPs) often involves toxic reagents or leads to particle agglomeration, and composite films usually exhibit insufficient mechanical properties. In this study, carboxymethyl chitosan (CMCS) was used as both a reducing and stabilizing agent to in situ reduce Ag^+^ under atmospheric-pressure oil-bath heating, yielding CMCS-Ag composites, which were then impregnated into carboxymethyl cellulose (CMC) films to prepare CMC-CMCS-Ag ternary composite packaging films. Under optimal conditions (102 °C, 6 h, 10 mM AgNO_3_, 0.05% CMCS), AgNPs were uniformly dispersed without aggregation. The tensile strength reached 125.45 MPa, and the inhibition zone diameters against *E. coli* and *S. aureus* were 14.1 mm and 14.7 mm, respectively. This work provides a facile strategy for high-performance antibacterial food packaging films.

## 1. Introduction

Conventional transparent plastic polymers, typified by polyethylene and polypropylene, are facing increasing restrictions in food packaging applications due to their inherent non-biodegradability, reliance on non-renewable resources, and contribution to environmental persistence and microplastic pollution [1,2]. Driven by escalating environmental concerns and increasingly stringent food safety regulations, the development of sustainable, biodegradable active packaging has become a research priority. In this context, biopolymers derived from natural polysaccharides—particularly cellulose-based materials—have garnered significant attention as promising alternatives in the food packaging industry [3].

As the most abundant natural biopolymer, cellulose offers distinct advantages, including widespread availability, excellent biocompatibility, and complete biodegradability. Carboxymethyl cellulose (CMC), a prominent cellulose derivative, exhibits superior water solubility, film-forming capability, and processing versatility. It is frequently fabricated into uniform, continuous films via solution casting for both passive and active packaging applications [4,5]. Furthermore, the abundance of hydroxyl and carboxyl groups along the CMC backbone facilitates interactions with various functional moieties [6,7], enabling the uniform incorporation and stable dispersion of active agents within the film matrix. These attributes render CMC a compelling matrix for functional packaging films [8]. However, pristine CMC films are limited by high hydrophilicity, poor moisture barrier properties, and a lack of intrinsic antimicrobial activity. These deficiencies significantly constrain their efficacy in long-term food preservation and active packaging scenarios [9]. Consequently, rational functional modification is imperative to enhance the comprehensive performance of CMC films, specifically by endowing them with robust antibacterial capabilities [10].

Silver nanoparticles (AgNPs) are widely recognized as potent antimicrobial agents. Attributable to their unique nanoscale effects and sustained ion-release behavior, AgNPs exhibit broad-spectrum inhibitory activity against bacteria, fungi, and viruses [11]. Currently, AgNP synthesis is primarily categorized into physical and chemical routes. Physical methods, such as arc discharge [12] and mechanical ball milling [13], yield high-purity AgNPs but often suffer from severe agglomeration due to the absence of stabilizers, which compromises antimicrobial efficacy. Moreover, these methods require sophisticated equipment, resulting in high costs and low efficiency. Conversely, chemical reduction [14] and electrochemical synthesis [15] can produce AgNPs with uniform particle sizes and colloidal stability; however, the requisite toxic reagents pose potential risks to human health and ecological safety. Therefore, utilizing natural biomaterials as dual reducing and stabilizing agents offers a sustainable and eco-friendly strategy for fabricating antimicrobial packaging materials.

Carboxymethyl chitosan (CMCS), a water-soluble chitosan derivative, possesses a wealth of hydroxyl, amino, and carboxyl functional groups. These active sites can effectively coordinate with silver ions, facilitating the green synthesis and stable immobilization of AgNPs under mild conditions. Extensive research confirms that CMCS not only promotes AgNP formation but also significantly augments the antibacterial performance of composite materials. For instance, Yang et al. achieved the photoreduction of silver ions utilizing the ɑ-hydroxyl groups of CMCS—without additional chemical reagents—to prepare multifunctional hydrogels with antibacterial and self-healing properties [16]. Similarly, Chen et al. employed CMC and starch as green reductants to synthesize 8–16 nm AgNPs, yielding composite films with a tensile strength of 9.8 MPa and potent antibacterial activity [17]. Furthermore, Zhao et al. developed chitosan-modified bacterial cellulose films via UV-induced photoreduction, which demonstrated broad-spectrum inhibition against both Gram-positive and Gram-negative bacteria, achieving an inhibition rate exceeding 80% within 48 h [18].

While CMCS has been widely explored for AgNP synthesis, its integration into CMC-based ternary systems remains largely unexplored. Existing literature predominantly focuses on binary CMCS-Ag composites, with a paucity of research on incorporating CMCS-mediated AgNPs into a CMC matrix to construct ternary CMC-CMCS–Ag composite films. Crucially, the interplay between AgNP synthesis conditions, microstructural characteristics, and the interfacial interactions among CMC, CMCS, and AgNPs—and their collective impact on the mechanical and antibacterial properties of the films—remains poorly understood. This lack of systematic exploration hinders the development of high-performance, multifunctional CMC-based active packaging. Therefore, it is essential to engineer novel ternary composite films and elucidate their structure-property relationships to meet the evolving demands of sustainable food packaging.

Herein, CMCS-Ag nanocomposites were synthesized using CMCS as a dual-function agent for both the reduction and stabilization of silver nanoparticles. Subsequently, ternary CMC-CMCS-Ag composite films were fabricated by immersing pre-formed, freeze-thawed CMC films into the CMCS-Ag dispersion. UV-Vis spectroscopy was employed to optimize the synthesis parameters of the CMCS-Ag nanocomposites. The microstructure and chemical composition of the resulting composites were characterized using TEM, SEM, XPS, and XRD. Furthermore, the mechanical properties and antibacterial efficacy of the ternary composite films were systematically evaluated to assess their potential for high-efficiency active food packaging applications.

## 2. Materials and Methods

### 2.1. Materials

Carboxymethyl cellulose II (AR, Macklin Biochemical Technology Co., Ltd., Shanghai, China) Carboxymethyl chitosan (AR, Macklin Biochemical Technology Co., Ltd., Shanghai, China). Silver nitrate (AR, Tianjin Oubokai Chemical Co., Ltd., Tianjin, China). Sulfuric acid (98%, Sinopharm Chemical Reagent Co., Ltd., Shanghai, China). Deionized water (AR, Laboratory Ultrapure Water System). Tryptone (AR, Beijing Solarbio Science & Technology Co., Ltd., Beijing, China). Yeast extract (AR, Beijing Solarbio Science & Technology Co., Ltd., Beijing, China). Agar powder (AR, Beijing Solarbio Science & Technology Co., Ltd., Beijing, China). Sodium chloride (AR, Tianjin Tianli Chemical Reagent Co., Ltd., Tianjin, China). *Escherichia coli* ATCC 35401 (Beijing Biobank Biotechnology Co., Ltd., Beijing, China). *Staphylococcus aureus* ATCC 25923 (Beijing Biobank Biotechnology Co., Ltd., Beijing, China).

### 2.2. Preparation of CMCS Solution

98 mL of deionized water was added to a beaker, followed by the addition of 2 g of CMCS powder under stirring. The mixture was stirred with a magnetic stir bar until complete dissolution, yielding a homogeneous 2% (*w*/*w*) CMCS solution. The solution was kept in a vial at 4 °C for further use.

### 2.3. Preparation of AgNO_3_ Solution

0.1699 g of AgNO_3_ was precisely weighed and dissolved in deionized water to obtain 100 mL of 10 mmol/L AgNO_3_ solution. The solution was kept in a brown vial at 4 °C in the dark until further use.

### 2.4. Preparation of CMCS-Ag Composites

CMCS solutions with volumes of 0.1 mL, 0.5 mL, 1.0 mL and 1.5 mL were used, and the corresponding volumes of deionized water were 17.4 mL, 17.0 mL, 16.5 mL and 16.0 mL, respectively. The corresponding volume of deionized water was added to a beaker, and different volumes of CMCS solution were dropped into water under uniform mixing. Afterwards, 2 mL of 10 mmol/L AgNO_3_ solution was added. A magnetic stir bar was placed in the beaker, and the mixture was magnetically stirred for 3 h. Subsequently, the mixed solution was transferred into a single-necked flask and reacted under stirring in an oil bath at 102 °C for 6 h. The solution gradually changed from transparent to yellowish-brown during the reaction. After completion of the reaction, the resulting solution was poured into a dialysis bag with a molecular weight cutoff of 3500 Da and dialyzed for five days to thoroughly remove residual Ag^+^. Finally, CMCS-Ag composite solutions with different CMCS contents (0.01%, 0.05%, 0.10%, 0.15%) were obtained.

To investigate the effect of synthesis conditions on the product, a single-factor experiment was conducted: with the CMCS content (0.05%) held constant, (1) reaction temperature was set to 100 °C, 101 °C, 102 °C, and 103 °C, while the reaction time was fixed at 6 h; (2) Reaction time: set to 2 h, 4 h, 6 h, and 8 h, with the reaction temperature fixed at 102 °C.

### 2.5. Preparation of Freeze-Thawed Carboxymethyl Cellulose Film (C0 and H0)

1.2 g of CMC powder was mixed with 58.8 mL deionized water and stirred at room temperature for 3 h until CMC was fully dissolved to obtain a 2 wt.% CMC stock solution. Two portions of the CMC solution were supplemented with 0 g and 2.55 g of 2 M sulfuric acid solution, respectively. Sulfuric acid protonates the carboxylate groups of CMC, which favors intermolecular hydrogen-bond formation in the following freeze–thaw process. 25 g of each mixed solution was cast into separate Petri dishes and treated in a vacuum oven at a constant vacuum pressure of 0.09 MPa for 12 h, followed by drying at 50 °C for 10 h. The dried samples were frozen at −17 °C for 8 h and then thawed at room temperature for 3 h, and this freeze–thaw cycle was repeated three times. All samples were finally dried in a vacuum oven at 50 °C for 6 h, and the resulting films were denoted as C0 and H0, respectively.

### 2.6. Preparation of CMC-CMCS-Ag Ternary Composite Film

The H0 film prepared in Section 2.5 was placed in a Petri dish. Then, 20 mL of the CMCS-Ag solution was added, and the film was immersed for 6 h. Subsequently, the dish was placed in an oven at 50 °C to dry off the water, yielding the CMC-CMCS-Ag ternary composite film.

### 2.7. Characterization

#### 2.7.1. Scanning Electron Microscopy (SEM)

Samples were freeze-dried, fractured, mounted on stubs, and sputter-coated with gold. SEM images were taken on an FEI Q45 instrument ((FEI Company, Hillsboro, OR, USA) equipped with an EDAX detector, using an accelerating voltage of 10 kV, following previously reported procedures with appropriate modifications [19].

#### 2.7.2. X-Ray Diffraction (XRD)

Dried samples were analyzed on an in situ Bruker diffractometer (Bruker AXS GmbH, Karlsruhe, Germany) with Cu Kα radiation (λ = 1.5406 Å). Data were collected over a 2θ range of 5–80° [20].

#### 2.7.3. X-Ray Photoelectron Spectroscopy (XPS)

XPS measurements were carried out on a Thermo Scientific K-Alpha spectrometer (Thermo Fisher Scientific, East Grinstead, UK) with a monochromatic Al Kα source (1486.6 eV). Constant analyzer energy mode was used, with pass energies of 150 eV for survey scans and 50 eV for high-resolution scans [21].

#### 2.7.4. UV–Vis Spectroscopy

UV–Vis spectra were recorded on an Agilent Cary 5000 spectrophotometer (Agilent Technologies, Santa Clara, CA, USA) over 200–800 nm, using deionized water as the blank [22].

#### 2.7.5. Transmission Electron Microscopy (TEM)

TEM was performed on an FEI Tecnai G2 F20 S-TWIN microscope (FEI Company, Hillsboro, OR, USA). A drop of sample suspension was placed on a 400-mesh copper grid, air-dried, stained with phosphotungstic acid, and dried again. Images were acquired at 100–300 kV [23].

#### 2.7.6. Fourier Transform Infrared (FTIR) Spectroscopy

FTIR spectra were collected on an INVENIO spectrometer (Bruker Optik GmbH, Ettlingen, Germany) over the range of 4000–400 cm^−1^ with a resolution of 4 cm^−1^ [20].

#### 2.7.7. Tensile Testing

Rectangular specimens (5 mm × 70 mm) were cut from each film according to ASTM D882 [24]. The specimens were clamped in the grips of a universal testing machine (AI-7000-NGD) with a 3000 N load cell. The initial grip separation was 20 mm, and the crosshead speed was 5 mm min^−1^. Tensile strength was measured under these conditions.

#### 2.7.8. Water Absorption

The water-absorption capacity of as-prepared samples was determined by a standard gravimetric method. Specimens were cut into uniform dimensions, and the initial dry mass ($m_{0}$) was accurately recorded. Each sample was fully immersed in distilled water at room temperature. The samples were taken out at preset soaking intervals (3 h, 6 h, 9 h, 12 h). Surface water was carefully wiped off with dry filter paper, and the wet mass ($m_{1}$) was measured [25]. The water absorption was calculated according to Equation (1):

(1) $$Water\;absorption\left(\%\right)=\frac{m_{1}-m_{0}}{m_{0}}*100$$

#### 2.7.9. Water Vapor Barrier Properties

The water vapor transmission rate of the films was measured using a Labthink W3/031 water vapor transmission rate tester (China) according to GB/T 1037-2021 [25] (gravimetric gain method). The measurement was performed at 23.0 ± 0.5 °C with a relative humidity of 50 ± 2% RH [26].

#### 2.7.10. Antibacterial Activities Testing

The liquid medium was prepared by dissolving 1.0 g peptone, 0.5 g yeast extract and 1.0 g NaCl in 100 mL deionized water, followed by autoclaving at 121 °C for 30 min. Solid medium was supplemented with 1.5 g agar; after sterilization and cooling to ~60 °C, 15–20 mL medium was poured into each sterile 90 mm Petri dish and stored at 4 °C. *E. coli* and *S. aureus* were cultured in liquid medium at 37 °C with shaking for 18–24 h, and the resultant suspensions were diluted with sterile PBS to 10^6^ CFU/mL [27]. Film samples were cut into 1 cm-diameter disks and disinfected under UV light. A 100 μL aliquot of bacterial suspension was spread evenly over solid medium, and the sterilized film disks were placed on the agar surface. After incubation at 37 °C for 24 h, plates were photographed and inhibition zone diameters were measured.

## 3. Results

### 3.1. Preparation of CMC-CMCS-Ag Ternary Composite Film

**Figure 1:**
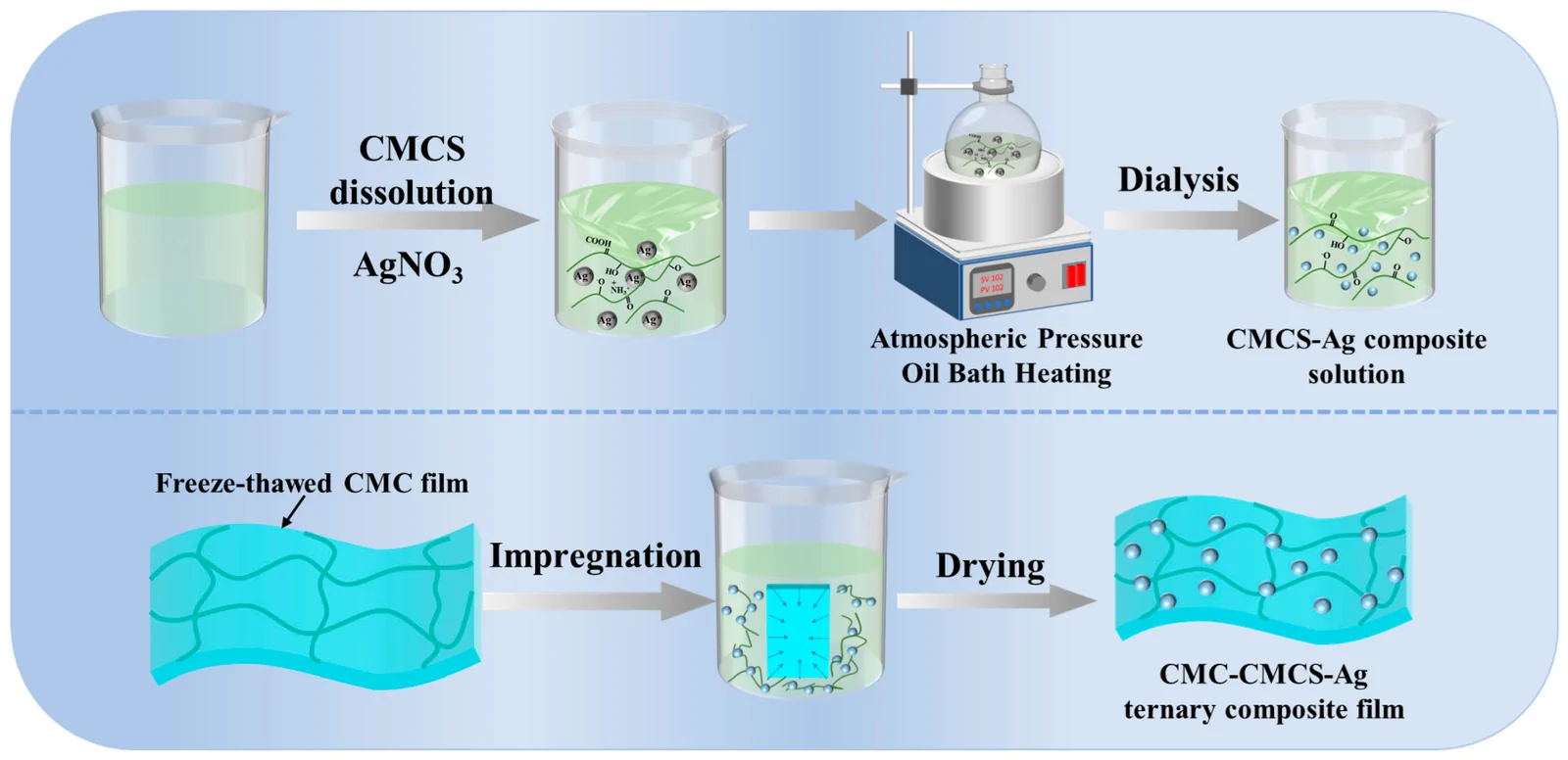
Schematic diagram of CMC-CMCS-Ag ternary composite film preparation.

The preparation process of the CMC-CMCS-Ag ternary composite film is illustrated in Figure 1. First, under atmospheric pressure, Ag^+^ was in situ reduced to AgNPs through a reaction in a high-temperature oil bath, thereby preparing a CMCS-Ag composite solution. Subsequently, the H0 film was fully immersed in this solution and dried to obtain the CMC-CMCS-Ag ternary composite film. During the process, CMCS served as both the reducing and stabilizing agent in the reaction system. Ag^+^ ions were electrostatically adsorbed onto the carboxyl groups of CMCS and then in situ reduced to form CMCS-Ag composites. No additional reducing or stabilizing agents were added throughout the reaction, making this an environmentally friendly and facile preparation method.

### 3.2. Optimization of CMCS-Ag Synthesis Conditions

**Figure 2:**
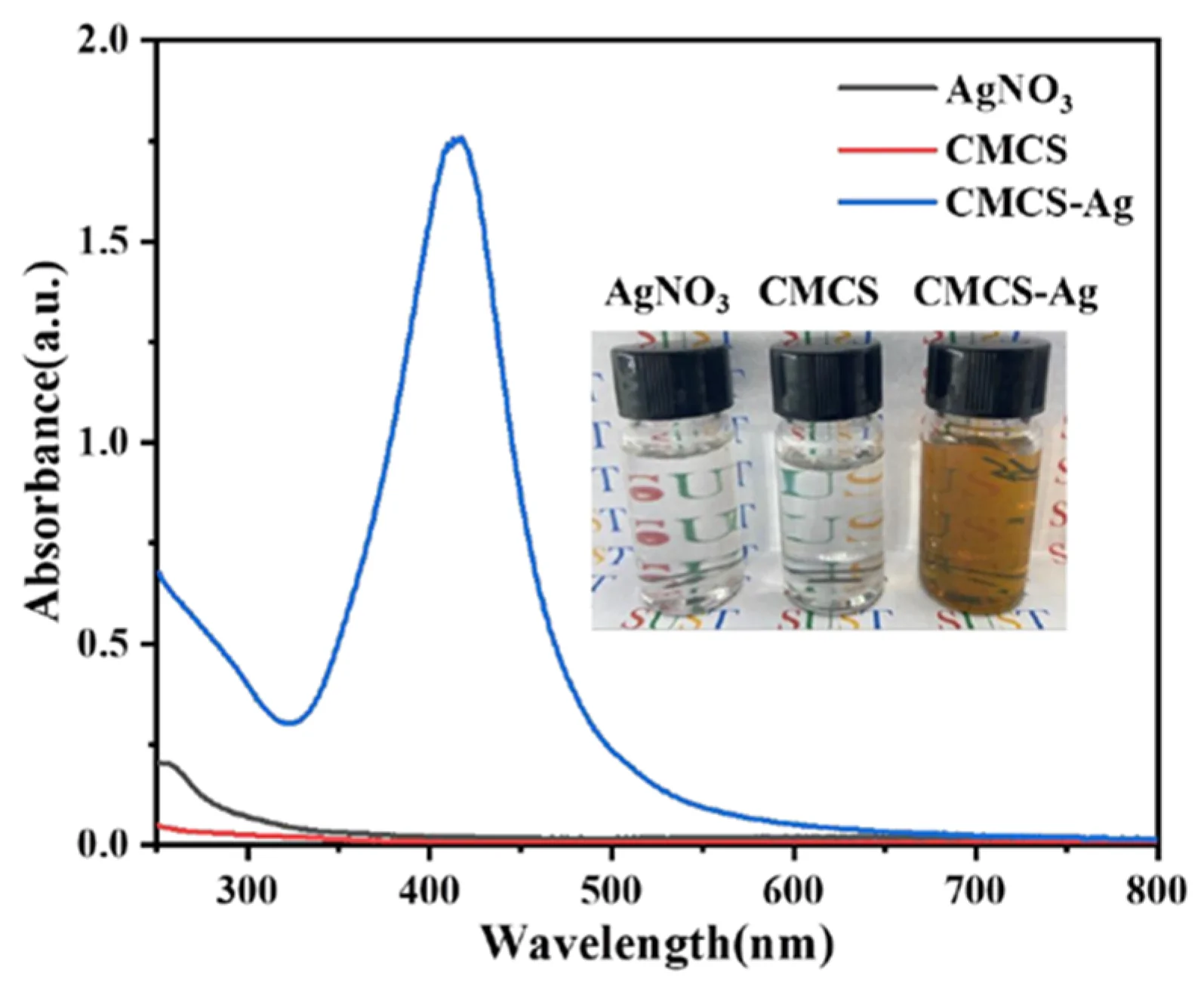
UV-Vis spectra of AgNO_3_, CMCS and CMCS-Ag composite solutions.

As shown in the optical photographs of the AgNO_3_, CMCS, and CMCS-Ag composite solutions in Figure 2, both the AgNO_3_ and CMCS solutions were initially transparent. During the synthesis of the CMCS-Ag composite, the solution color gradually changed from transparent to yellow and finally to brown, indicating the formation of AgNPs with different particle sizes. UV-Vis spectroscopy was also employed to monitor the formation process of AgNPs [28,29]. As can be seen from Figure 2, neither the CMCS solution nor the AgNO_3_ solution exhibited any absorption peak in the range of 250–800 nm. In contrast, the CMCS-Ag solution showed a characteristic absorption peak of AgNPs at around 404 nm, confirming the presence of AgNPs and the successful formation of the CMCS-Ag composite [30].

#### 3.2.1. Effect of CMCS Concentration on the Synthesis of CMCS-Ag Composites

**Figure 3:**
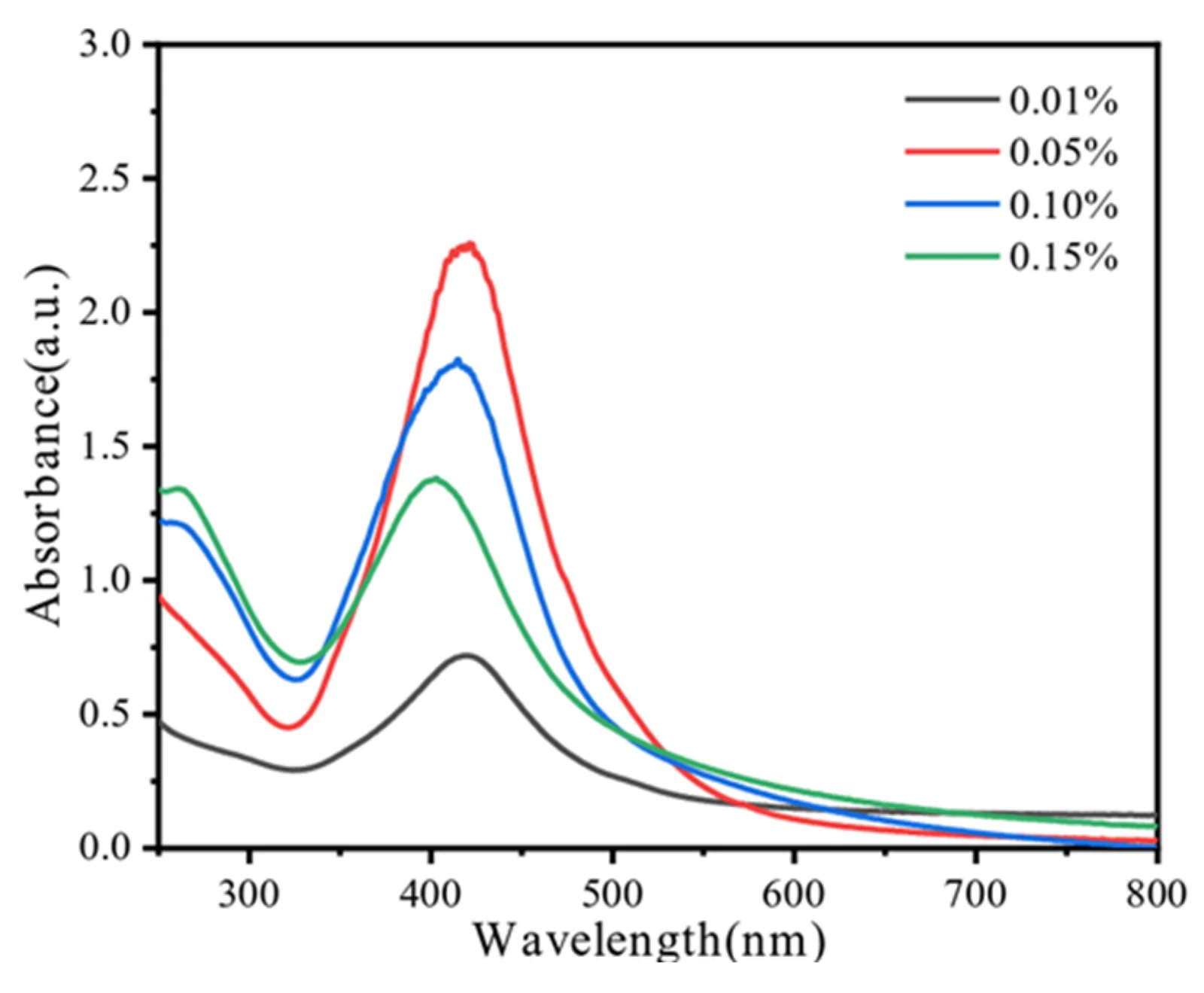
UV-Vis spectra of CMCS-Ag composites at different concentrations of CMCS.

The size and size distribution of AgNPs can be inferred from changes in their UV-Vis absorption peaks. Figure 3 shows the effect of different CMCS mass fractions on the synthesis of CMCS-Ag composites, with a reaction temperature of 102 °C, reaction time of 6 h, and AgNO_3_ concentration of 10 mM. As shown in the figure, when the CMCS mass fractions were 0.01%, 0.05%, 0.10%, and 0.15%, characteristic absorption peaks of AgNPs appeared at around 404 nm. The intensity of the characteristic peak first increased and then decreased. The peak height corresponds to the amount of AgNPs generated [31]. At a CMCS mass fraction of 0.01%, the absorption peak exhibited a broad full width at half maximum (FWHM) and low intensity, which may be attributed to the insufficient amount of CMCS acting as a both reducing and stabilizing agent, leading to incomplete reduction of Ag^+^ and fewer AgNPs [32]. When the CMCS mass fraction reached 0.05%, the characteristic absorption peak reached its maximum, indicating the highest yield of AgNPs under this condition. Moreover, the FWHM of the peak was narrow, suggesting a uniform size distribution and relatively small AgNPs, which are likely to exhibit good antibacterial activity [33]. At CMCS mass fractions of 0.10% and 0.15%, the peak height decreased and the FWHM broadened, indicating non-uniform size distribution of the generated AgNPs. In summary, 0.05% was selected as the optimal CMCS mass fraction for the preparation of CMCS-Ag composites.

#### 3.2.2. Effect of Reaction Temperature on CMCS-Ag Composites

**Figure 4:**
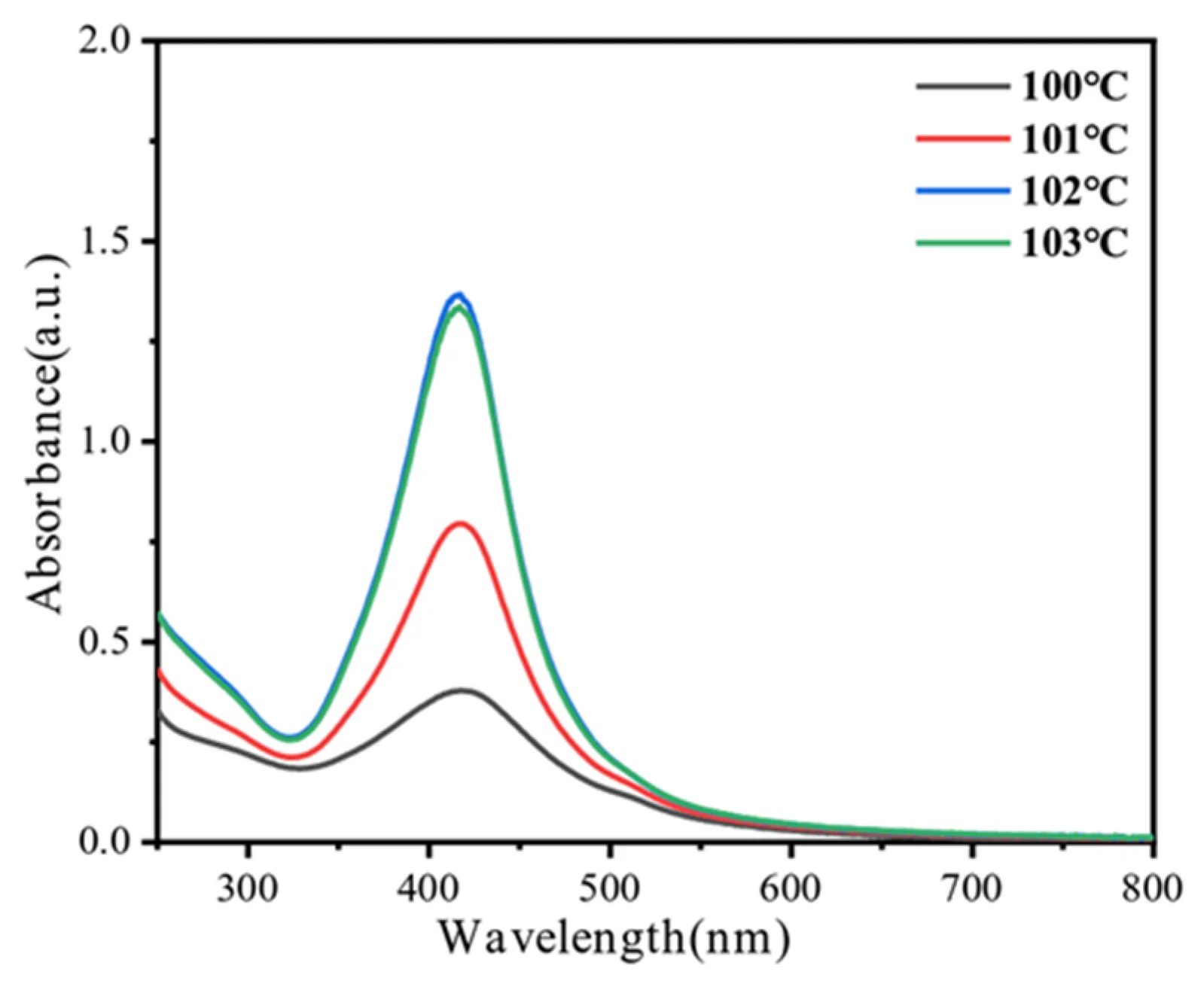
UV-Vis spectra of CMCS-Ag composites at different temperatures.

Temperature has a pronounced effect on chemical reactions, and variations in reaction temperature can greatly influence the reaction rate [34,35,36,37]. Figure 4 shows the UV-Vis spectra of CMCS-Ag composites synthesized at different reaction temperatures (100 °C, 101 °C, 102 °C, and 103 °C), while keeping other reaction conditions constant (0.05% CMCS mass fraction, 6 h reaction time, 10 mM AgNO_3_ concentration). As can be seen from the figure, all spectra exhibited a characteristic absorption peak of AgNPs at around 404 nm, indicating the formation of AgNPs in all cases. At 100 °C, the peak showed a broad full width at half maximum (FWHM) and low intensity, suggesting insufficient reaction temperature and a lower yield of AgNPs. As the temperature increased, the characteristic peak intensity gradually rose. However, at 103 °C, the peak intensity decreased. This may be attributed to the rapid formation rate of AgNPs at higher temperatures, which prevented CMCS from providing adequate stabilization, leading to partial aggregation of AgNPs and a consequent reduction in the characteristic peak intensity [38,39]. In summary, 102 °C was selected as the optimal reaction temperature for the preparation of CMCS-Ag composites.

#### 3.2.3. Effect of Reaction Time on CMCS-Ag Composites

**Figure 5:**
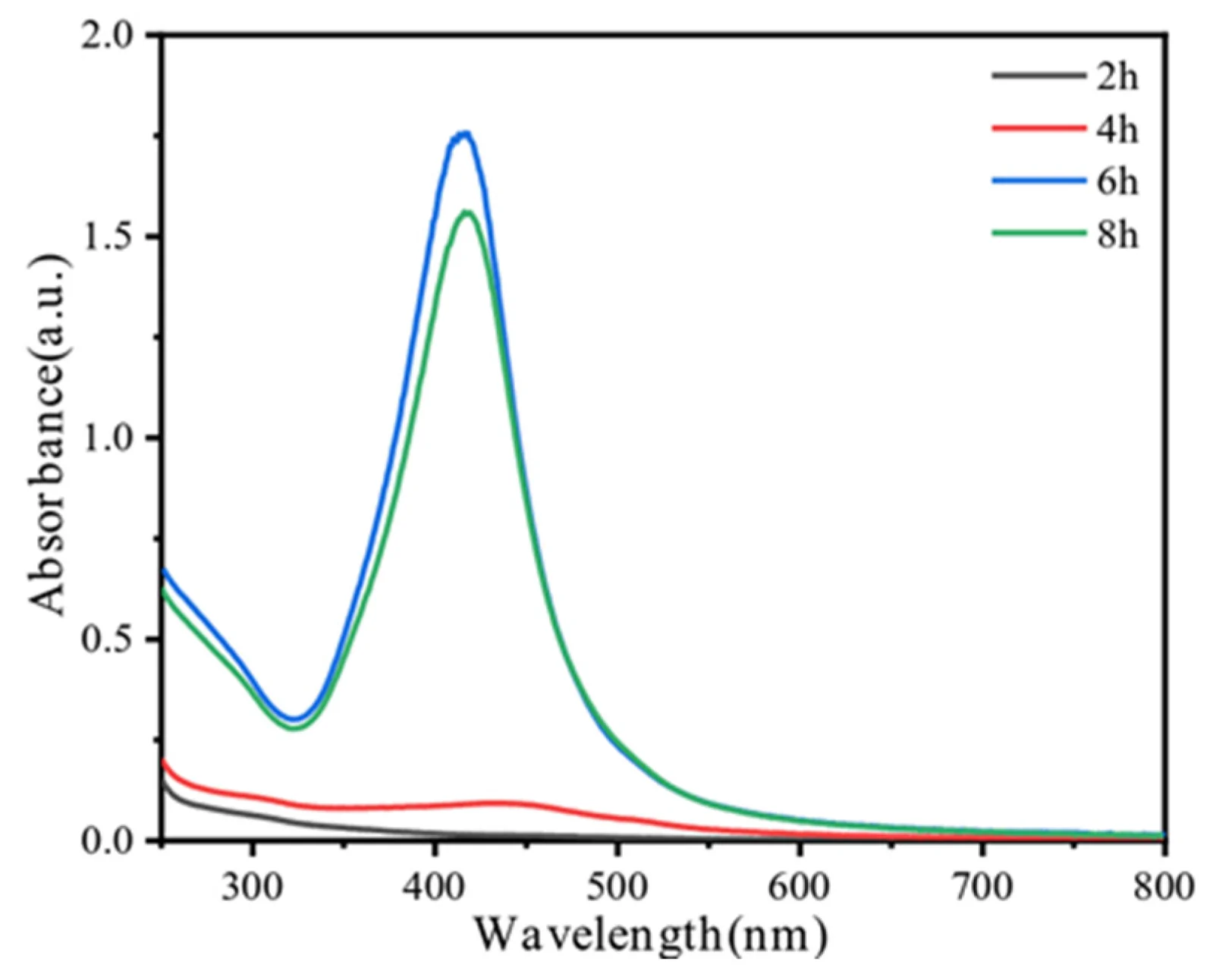
UV-Vis spectra of CMCS-Ag composites at different times.

Figure 5 shows the UV-Vis spectra of CMCS-Ag composites synthesized at different reaction times. Other conditions were kept constant (0.05% CMCS mass fraction, reaction temperature 102 °C, 10 mM AgNO_3_ concentration). At reaction times of 6 h and 8 h, distinct characteristic absorption peaks of AgNPs appeared at around 404 nm, indicating the presence of AgNPs in the composites. When the reaction time was 2 h, no characteristic peak was observed, suggesting insufficient reaction time for CMCS to reduce Ag^+^, and thus no AgNPs were formed. At 4 h, a weak and broad absorption peak appeared, indicating the onset of AgNPs formation but with low yield and non-uniform distribution [40]. At 8 h, the intensity of the characteristic peak decreased. This can be explained by the continued nucleation and aggregation of small AgNPs after a sufficient amount had been generated, leading to a reduction in the number of nanoparticles in the system and a corresponding decrease in absorbance [41]. In summary, 6 h was selected as the optimal reaction time for the preparation of CMCS-Ag composites.

In summary, after investigating the synthesis conditions for CMCS-Ag composites, the optimal parameters were determined as follows: CMCS mass fraction of 0.05%, AgNO_3_ concentration of 10 mM, reaction temperature of 102 °C, and reaction time of 6 h. CMCS-Ag composites prepared under these conditions were used for subsequent studies.

### 3.3. Morphological Characterization of CMCS-Ag Composites

**Figure 6:**
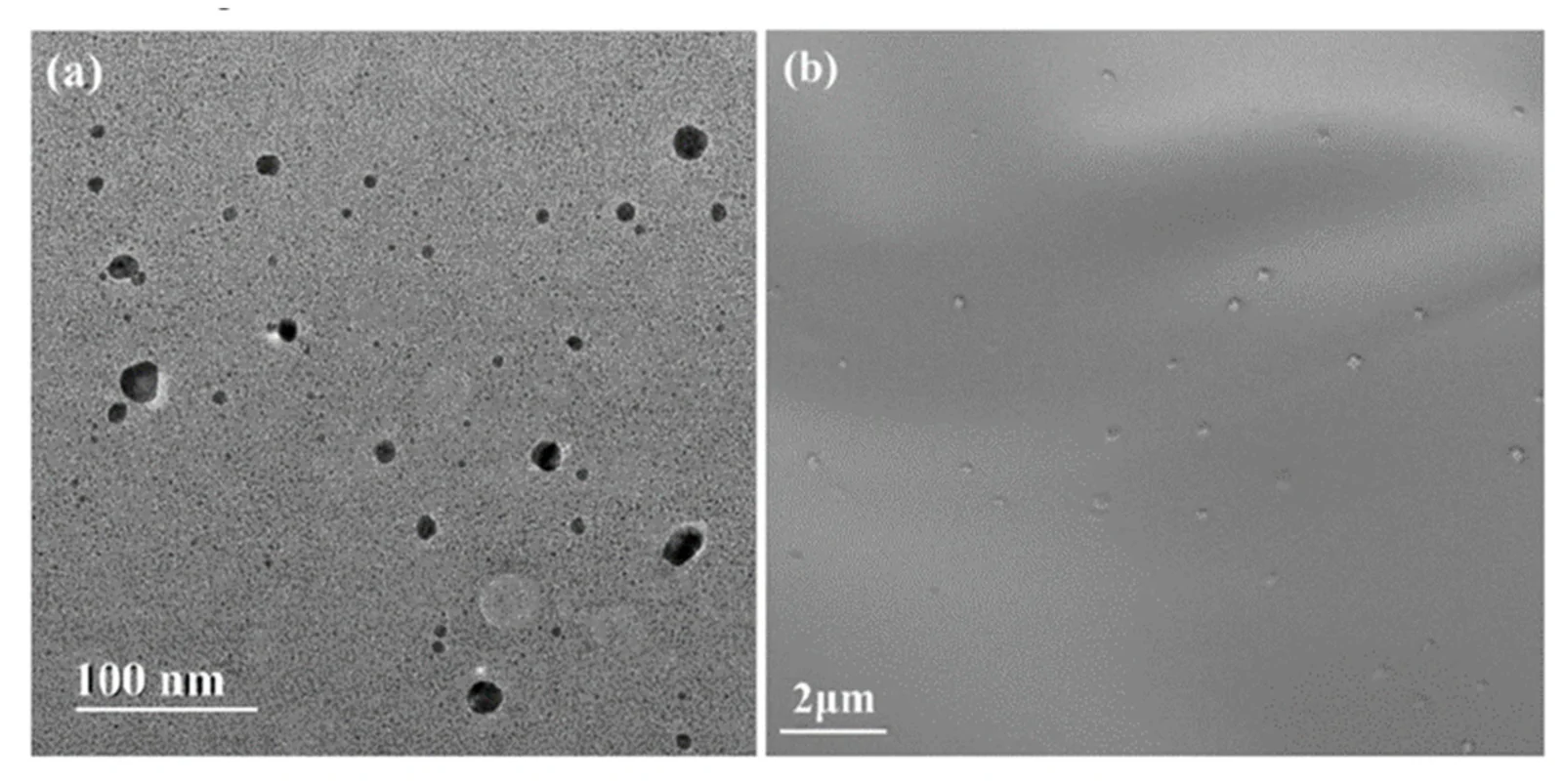
(**a**) TEM image of CMCS-Ag; (**b**) SEM image of CMCS-Ag.

Figure 6a shows TEM images of the CMCS–Ag composite, where spherical AgNPs are clearly visible. The particles are mostly well dispersed, with no obvious aggregation except for a few local spots of slight clustering. This uniform dispersion is mainly due to CMCS playing two roles in the reaction: reducing Ag^+^ and stabilizing the formed AgNPs against agglomeration [42]. The minor clustering likely arises from chain entanglement among polymer molecules. Figure 6b presents SEM images of CMCS–Ag, where fine protrusions are evenly distributed on the matrix surface, confirming that AgNPs were successfully reduced and the CMCS–Ag composite was formed. The AgNPs obtained here range from 10 to 40 nm, comparable to those reported for similar CMCS–Ag systems, though with a somewhat broader size distribution [43]. This broadening is likely due to the relatively long reaction time (6 h at 102 °C in an oil bath), which promotes particle ripening and growth. Unlike strong reducing systems such as NaBH_4_ [44], the present method uses no external toxic reductants and is thus greener, but it does give a wider particle size distribution. This could be improved in future work by optimizing the reaction conditions.

### 3.4. Characterization of the Particle Size Distribution of CMCS-Ag

**Figure 7:**
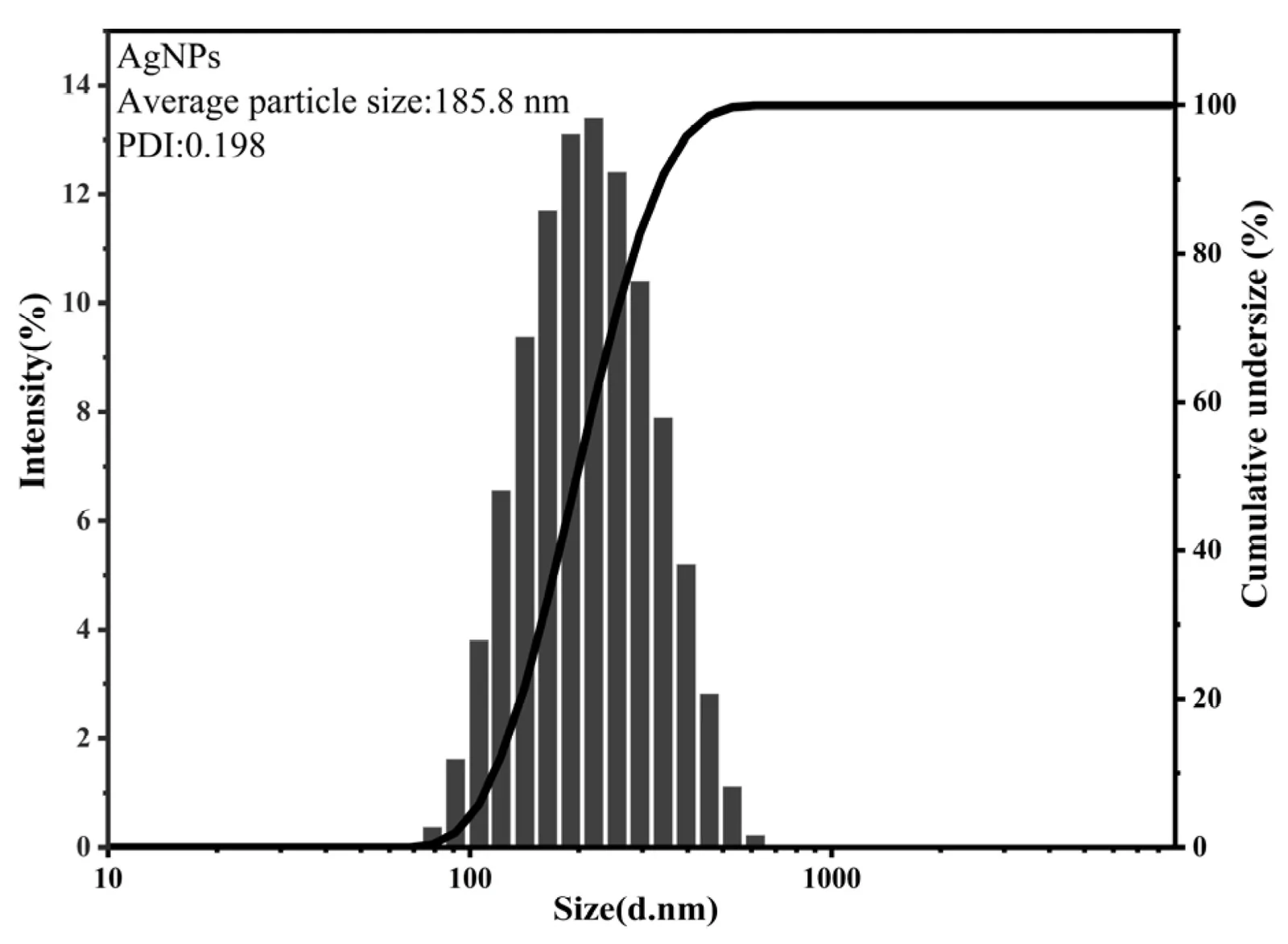
Particle-size distribution histogram of the CMCS-Ag colloidal solution, along with the average particle size and polydispersity index (PDI).

A nanoparticle size and zeta potential analyzer was used to measure the particle size distribution of silver nanoparticles in the CMCS-Ag colloidal solution. As shown in Figure 7, the results showed that the average particle size of the silver nanoparticles was 185.8 nm, with a PDI of 0.198. A PDI value of less than 0.2 indicates that the synthesized silver nanoparticles have a narrow size distribution, good dispersion, and no significant agglomeration. The statistical size distribution is primarily concentrated in the 100–300 nm range. These results suggest that, under the current synthesis conditions, CMCS acts as an effective stabilizer to suppress excessive agglomeration of silver nanoparticles, resulting in a silver nanoparticle colloid with good dispersion.

### 3.5. X-Ray Diffraction (XRD) Analysis of CMCS-Ag Composites

**Figure 8:**
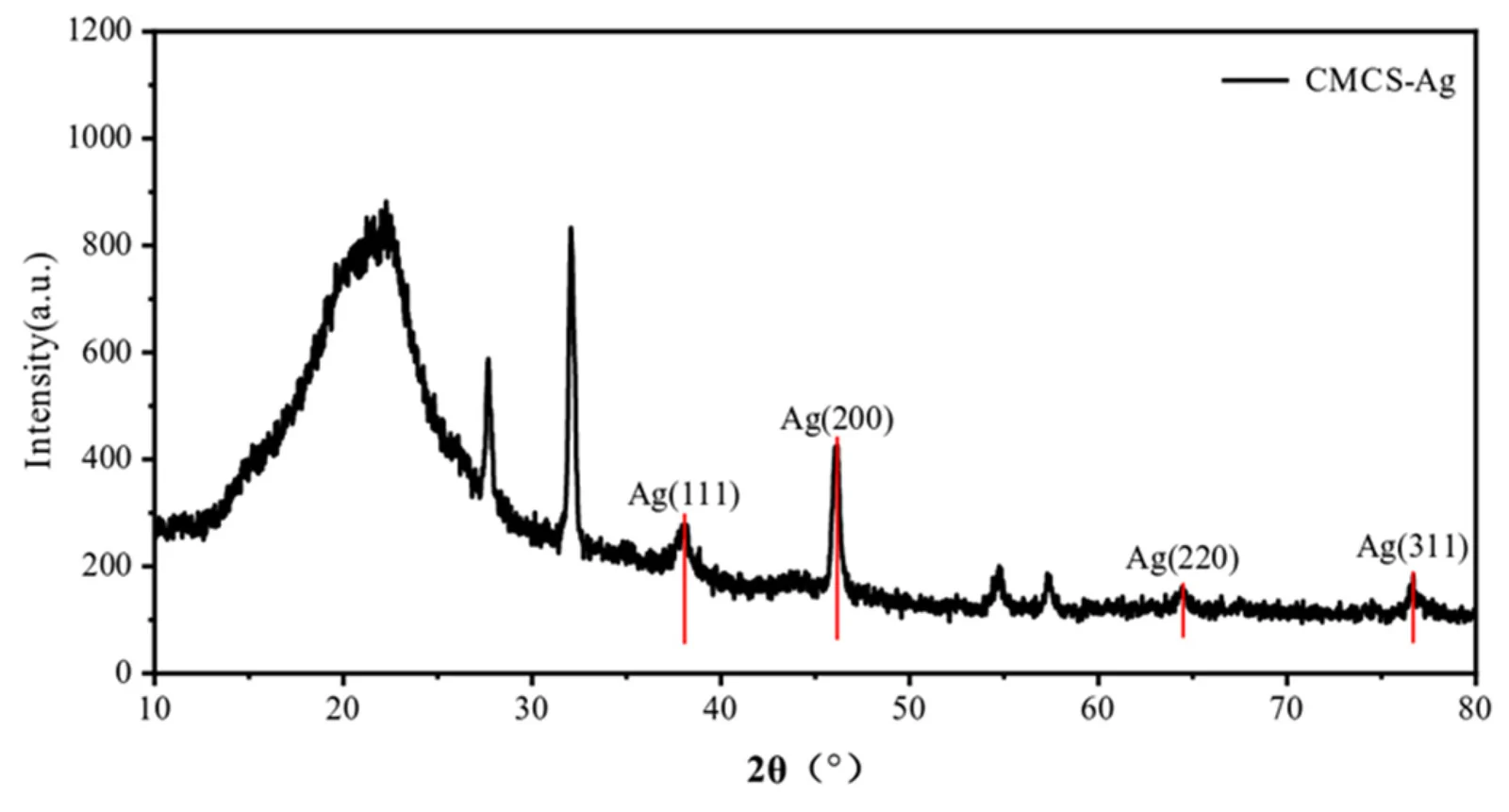
X-ray diffraction pattern of CMCS-Ag composite material.

XRD analysis confirmed AgNP formation in the CMCS–Ag composite. Figure 8 shows the diffraction pattern of the sample. Four characteristic peaks of metallic silver appear at 2θ = 38.1°, 44.3°, 64.4°, and 77.4°, corresponding to the (111), (200), (220), and (311) planes, respectively. These match the standard pattern for silver, confirming that CMCS reduces Ag^+^ to AgNPs in situ. The sharpness of these peaks indicates good [45]. A broad signal around 2θ ≈ 20° comes from the amorphous CMCS matrix. Several weak, sharp peaks are also visible near 27°, 32°, 55°, and 56°. Based on previous XRD studies on Ag/Ag_2_O systems, these likely arise from trace amounts of Ag_2_O or other silver-containing by-products [46]. This could be due to incomplete reduction of Ag^+^ during the oil-bath treatment or partial surface oxidation of AgNPs during storage. Overall, although the system converts most Ag^+^ to metallic silver, further optimization of reaction conditions and storage methods is still needed.

### 3.6. X-Ray Photoelectron Spectroscopy (XPS) Analysis of CMCS-Ag Composites

**Figure 9:**
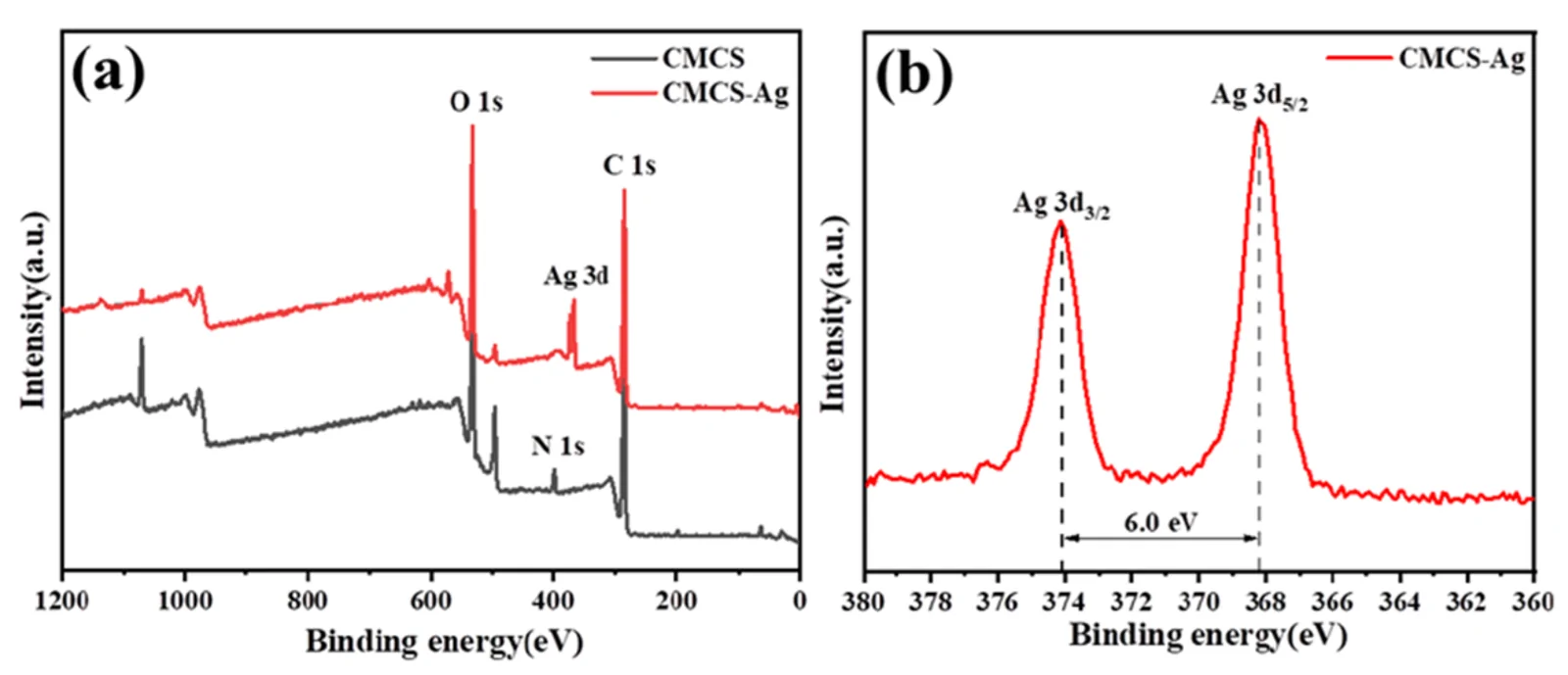
(**a**) X-ray photoelectron spectra of CMCS-Ag composite material and CMCS; (**b**) XPS spectra of Ag 3d region in CMCS-Ag composite material.

Figure 9a shows the XPS survey spectra of pure CMCS and the CMCS–Ag composite, providing a direct view of their surface elemental compositions. A clear Ag 3d signal appears in the CMCS–Ag sample but not in pure CMCS, confirming that silver was successfully introduced into the CMCS matrix. Two sharp peaks are seen in the pure CMCS spectrum around 500 eV and 1100 eV. These do not match the characteristic peaks of C, N, or O in CMCS and are thus likely due to instrument background or accessory signals during measurement. Figure 9b presents the high-resolution Ag 3d XPS spectrum of the CMCS–Ag composite. The Ag 3d region shows a typical spin–orbit doublet, with peaks at 368.12 eV and 374.12 eV, corresponding to Ag 3d_5_/_2_ and Ag 3d_3_/_2_, respectively, with a splitting of 6.0 eV. According to the literature, zero-valent silver typically shows Ag 3d_5_/_2_ and Ag 3d_3_/_2_ peaks at around 368 eV and 374 eV, with a spin–orbit splitting of about 6.0 eV [47,48]. The values obtained here are in good agreement with these standard features of metallic silver, further confirming that Ag+ was effectively reduced to AgNPs. Moreover, no obvious shift in binding energy is observed compared with reported values for metallic silver, suggesting that coating with CMCS does not significantly affect the surface electronic structure of the silver particles.

### 3.7. Morphological Characterization of CMC–CMCS–Ag Ternary Composite Film

**Figure 10:**
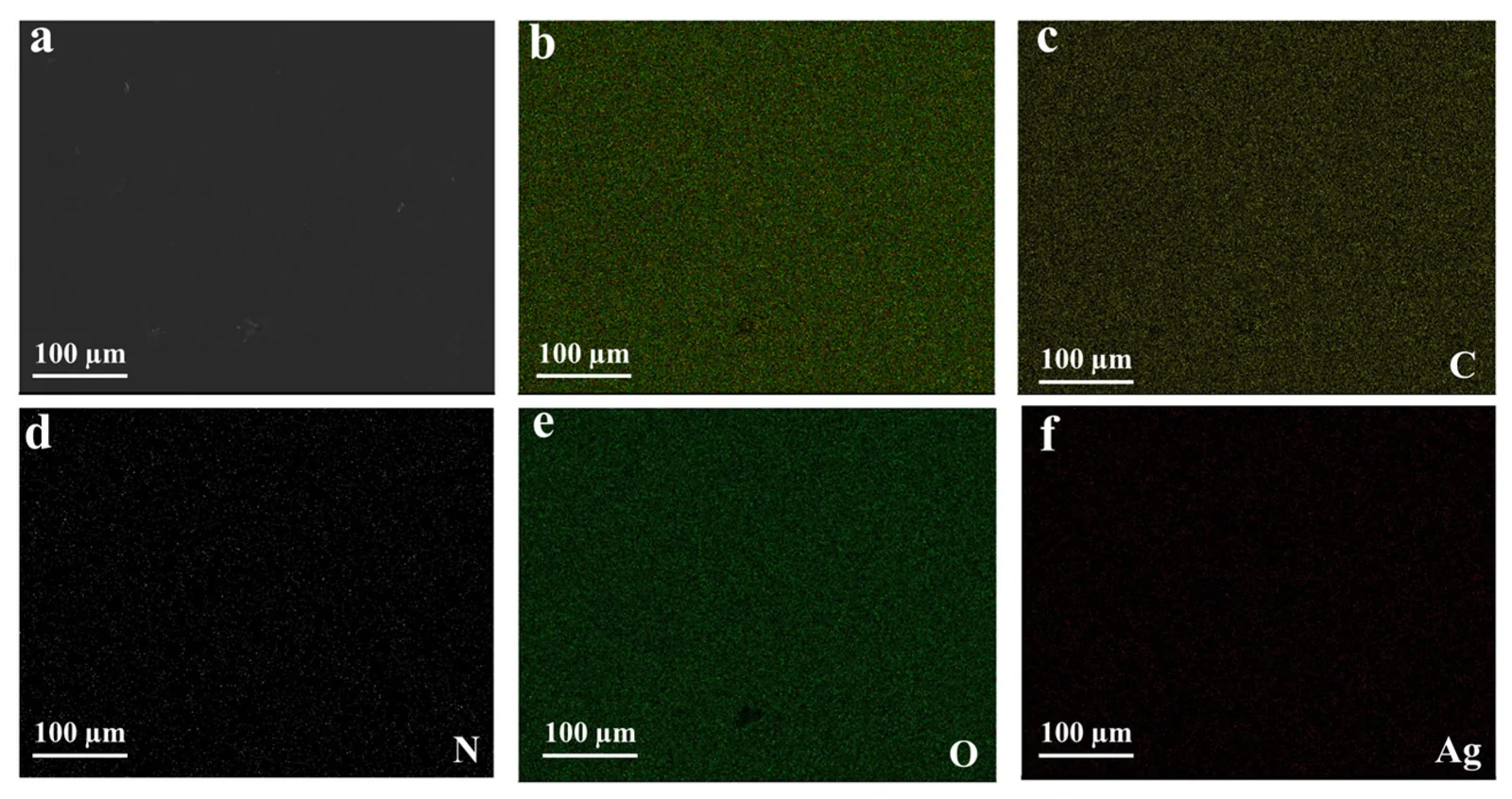
SEM-EDS characterization of CMC-CMCS-Ag ternary composite film. (**a**) SEM surface morphology of the film; (**b**) full-range EDS survey spectrum; elemental mapping distribution of (**c**) C, (**d**) N, (**e**) O, and (**f**) Ag.

Figure 10 shows the SEM-EDS results for the CMC–CMCS–Ag ternary composite film. Figure 10a depicts the SEM morphology of the film surface. The EDS full spectrum in Figure 10b indicates that the sample contains elements such as C, O, N, and Ag. The results of the elemental mapping scan indicate that C and O are uniformly distributed throughout the film, originating from the polysaccharide backbones of CMC and CMCS; N is also uniformly dispersed, suggesting that CMCS has been uniformly incorporated into the film; and Ag is similarly dispersed throughout the entire field of view, with no obvious aggregation or localized enrichment. These observations directly demonstrate that, following the impregnation treatment, silver nanoparticles are uniformly loaded into the CMC–CMCS–Ag film matrix.

### 3.8. FTIR Analysis of the CMC–CMCS–Ag Ternary Composite Film

**Figure 11:**
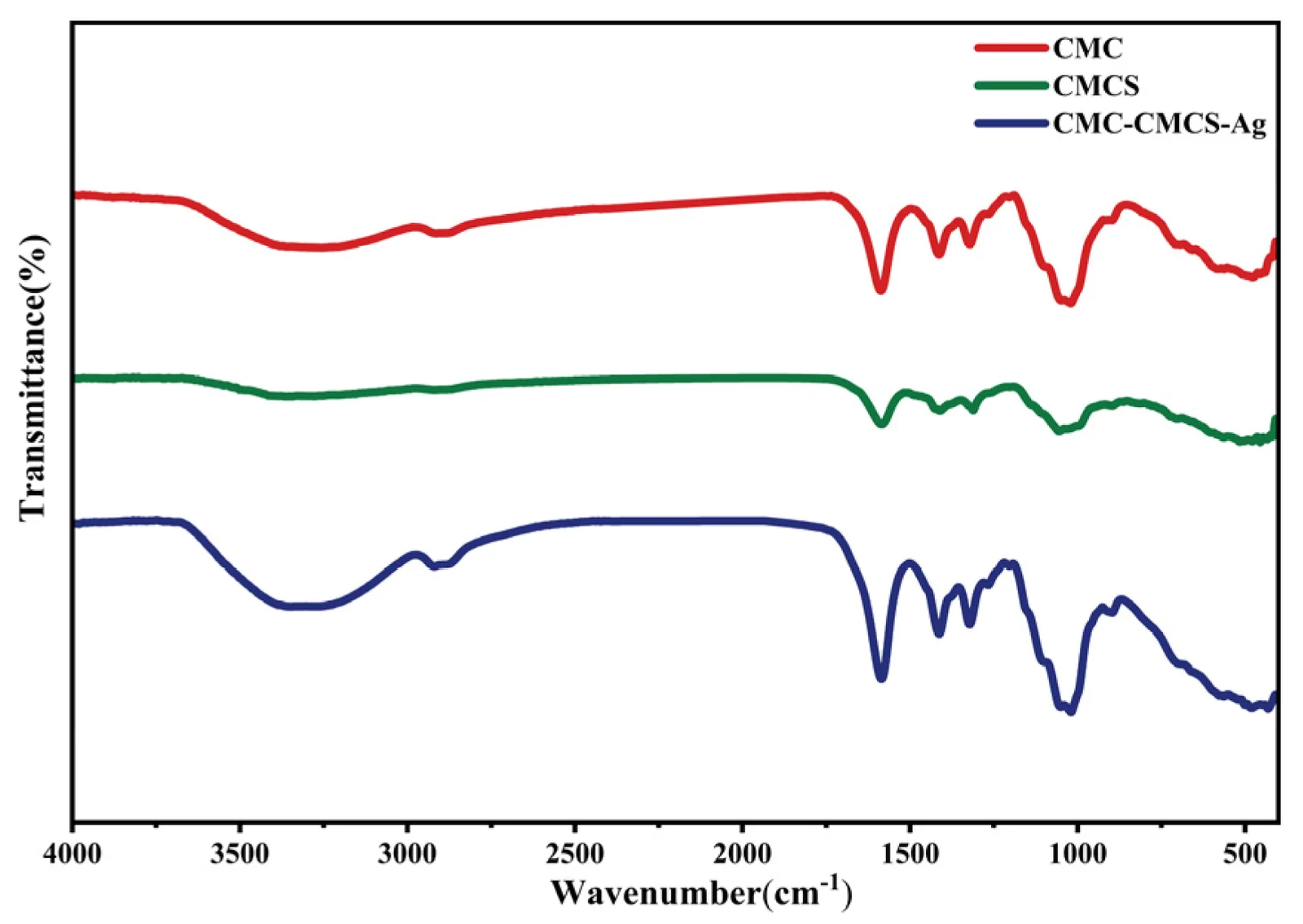
FTIR spectra of CMC, CMCS and CMC–CMCS–Ag ternary composite film.

As shown in Figure 11, the spectrum of neat CMC shows a broad band at 3200–3600 cm^−1^, arising from O–H stretching of hydroxyl groups. The peaks near 1600–1640 cm^−1^ and 1420 cm^−1^ correspond to asymmetric and symmetric stretching of carboxylate (–COO-), respectively. The several bands in the 1000–1100 cm^−1^ region are attributed to C–O–C and C–O stretching of the pyranose ring skeleton, which are characteristic fingerprint signals of CMC. For neat CMCS, besides the polysaccharide backbone absorptions, the broad band at 3200–3600 cm^−1^ includes contributions from both O–H and N–H stretching. In the 1500–1600 cm^−1^ region, overlapping signals from N–H bending and carboxylate stretching are seen, reflecting the coexistence of hydroxyl, amino, and carboxyl groups in CMCS. Compared with the single components, the ternary composite shows no new covalent peaks, indicating that no significant new covalent structures formed during blending. However, the O–H/N–H band at 3200–3600 cm^−1^ becomes noticeably wider, and the carboxylate band at 1600–1640 cm^−1^ changes in both intensity and shape. These changes arise from intermolecular interactions between CMC and CMCS: hydrogen bonds between carboxyl and hydroxyl groups on CMC and hydroxyl and amino groups on CMCS, along with electrostatic attraction between negatively charged carboxylate (–COO-) and protonated amino groups (–NH_3_^+^). Together, these two effects alter the local chemical environment of the functional groups, which is reflected in the FTIR peak shape and intensity changes. AgNPs themselves do not absorb in the IR region, but their presence may further perturb the surrounding polysaccharide environment and contribute synergistically to the observed spectral changes.

### 3.9. XRD and XPS Analysis of the CMC–CMCS–Ag Ternary Composite Film

**Figure 12:**
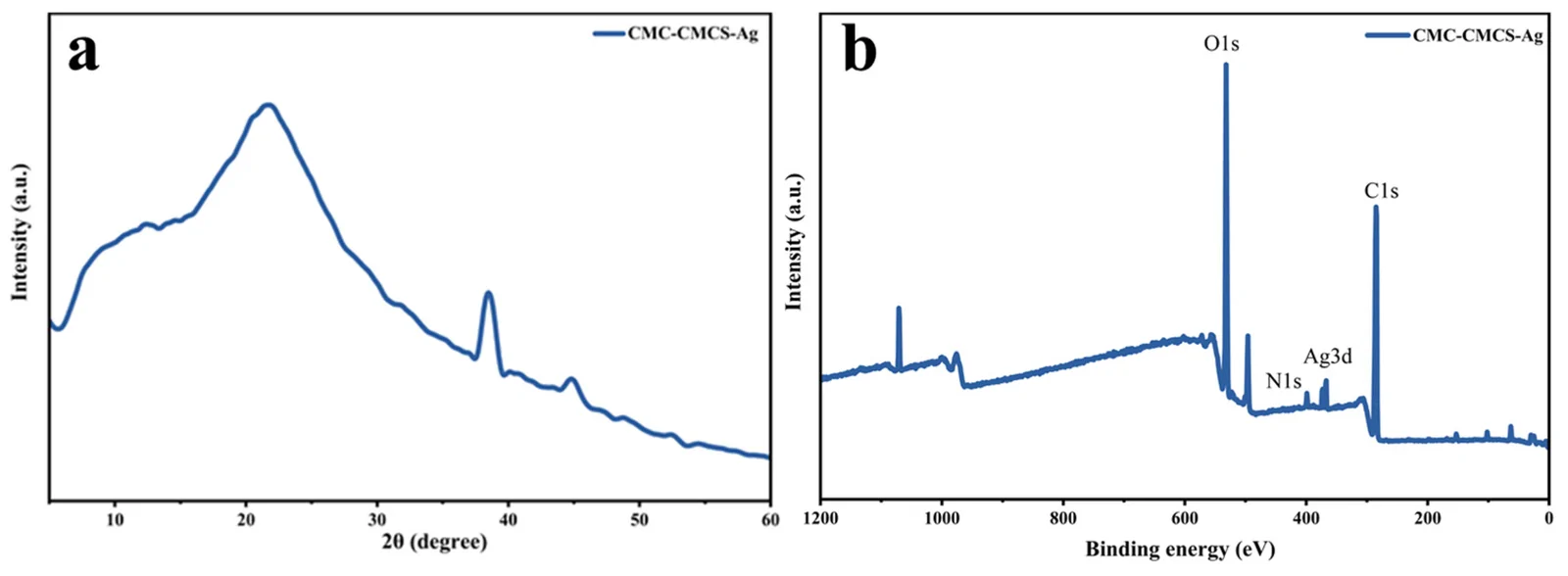
(**a**) XRD spectra of CMC–CMCS–Ag ternary composite film; (**b**) XPS spectra of CMC–CMCS–Ag ternary composite film.

Figure 12a shows the XRD pattern of the CMC–CMCS–Ag ternary composite film. A broad diffuse peak appears at 2θ = 22°, which is typical for carboxymethyl chitosan and reflects the amorphous structure of the CMC and CMCS polymers. A distinct diffraction peak is observed at 2θ = 38.2°, corresponding to the (111) plane of metallic silver, confirming that AgNPs were successfully incorporated into the film. The other characteristic peaks of silver are not clearly seen, most likely because of the low AgNP content and small crystallite size, which cause their signals to be masked by the strong background from the polysaccharide matrix.

Figure 12b shows the full XPS spectrum of the CMC–CMCS–Ag ternary composite film. Characteristic signals of C 1s, O 1s, and N 1s are visible in the spectrum; the C and O signals originate from the polysaccharide backbones of CMC and CMCS, while the presence of the N 1s peak indicates that CMCS has been successfully incorporated into the film. A distinct Ag 3d doublet appears in the binding energy range of 360–380 eV, which directly confirms that silver nanoparticles have been loaded onto the film surface following the impregnation treatment. Furthermore, the sharp minor peaks near 500 eV and 1100 eV in the spectrum are satellite peaks generated by the Al Kα excitation source; these are part of the instrument’s background signal and are unrelated to the sample itself.

### 3.10. Mechanical Properties of CMC-CMCS-Ag Ternary Composite Films

**Figure 13:**
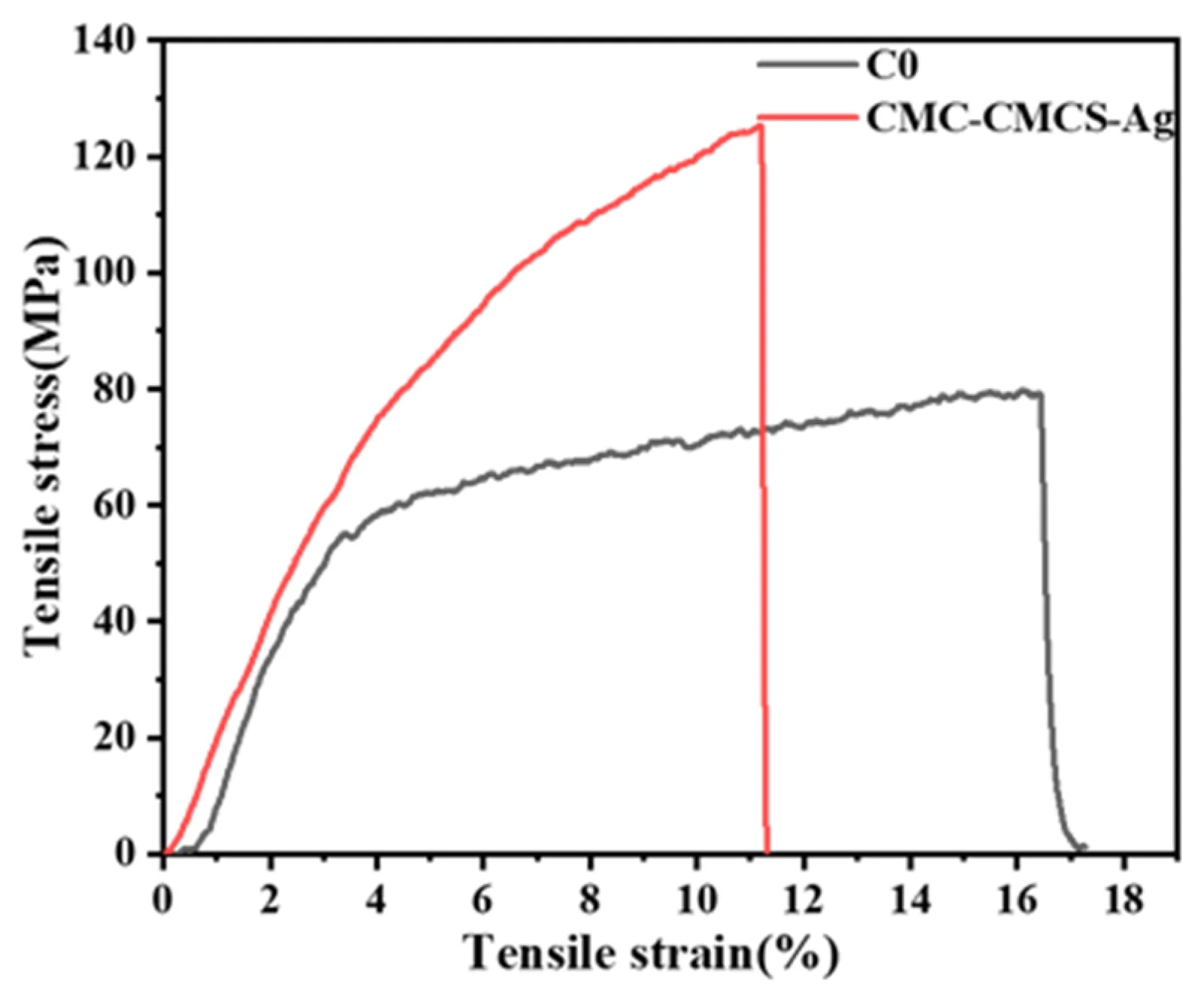
Stress–strain curves of C0 thin film and CMC-CMCS-Ag ternary composite thin film.

Mechanical performance is a key indicator of practicality for food packaging films. Films must be strong enough to resist external damage and ensure package integrity and protection. Figure 13 presents the stress–strain curves of neat CMC film (C0) and the CMC-CMCS-Ag ternary composite film. The composite shows a tensile strength of 125.45 MPa, a 57.32% increase over the 79.74 MPa of pure CMC—a substantial improvement. This enhancement arises from synergistic effects at multiple structural levels. Acid-assisted freeze–thaw treatment protonates the carboxyl groups on CMC, exposing more hydroxyl sites that form stronger, less reversible hydrogen bonds. Subsequent drying and dehydration shorten the distances between polymer chains, densifying the hydrogen-bond network [49]. Meanwhile, CMCS disperses evenly in the CMC matrix, giving good interfacial compatibility, and mild electrostatic interactions further stabilize the overall structure. Together, these factors greatly boost mechanical properties. Most current studies achieve higher strength by adding rigid nanofillers, but this often reduces flexibility, raises costs, and introduces aggregation defects. In contrast, our approach uses simple physical modification and polymer blending to obtain a notable strengthening effect, with easier processing and a more uniform film structure—offering clear advantages for packaging materials [50].

### 3.11. Analysis on Water Absorption and Water Vapor Barrier Properties of CMC-CMCS-Ag Ternary Composite Films

**Figure 14:**
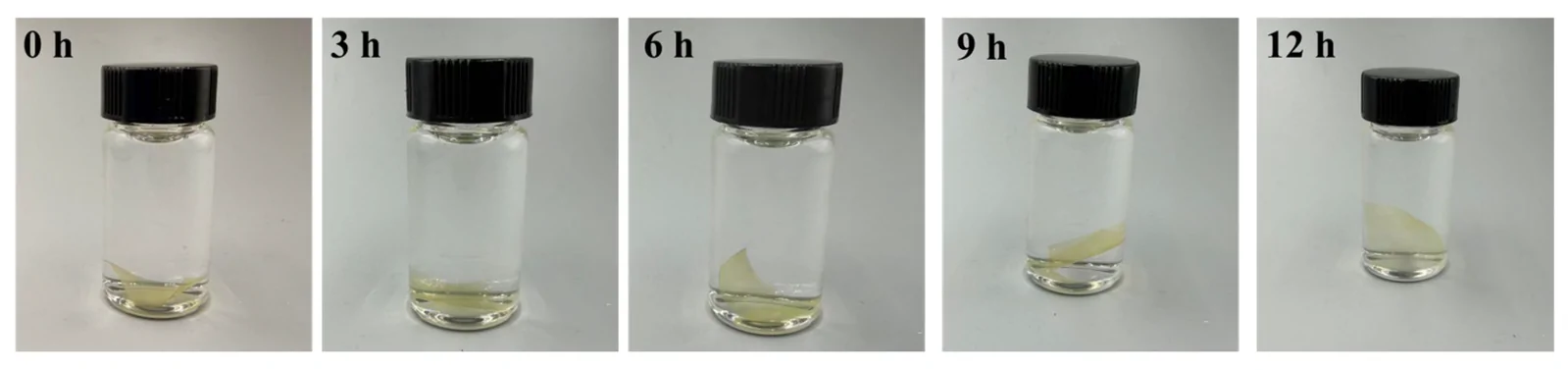
Digital photographs of CMC-CMCS-Ag ternary composite films after soaking for 0 h, 3 h, 6 h, 9 h and 12 h.

Figure 14 shows the changes in the appearance of the film after being immersed in deionized water for varying periods of time. Table 1 lists the corresponding water absorption rates, and Table 2 shows that the water vapor transmission rate was measured to be 48.65 g/(m^2^·24 h). During the first 3 h of soaking, the film absorbed water rapidly, reaching a water absorption rate of 88.18%. This is attributed to the presence of a large number of hydrophilic groups, such as hydroxyl and carboxyl groups, in both the CMC matrix and the CMCS introduced during impregnation. Subsequently, the rate of water absorption slowed, with water absorption rates at 6, 9, and 12 h reaching 93.24%, 94.93%, and 98.31%, respectively, gradually approaching saturation. Although the film exhibited strong hydrophilicity, it remained intact after 12 h of soaking, showing no signs of disintegration or dissolution. This was primarily due to the acid-assisted freeze–thaw treatment, which had pre-established a dense hydrogen-bond network within the CMC, forming a stable skeletal structure [51]; simultaneously, additional hydrogen bonds formed between the CMCS introduced into the film via impregnation and the CMC, while the silver nanoparticles also served as physical cross-links. These factors collectively inhibit the excessive dissolution of polysaccharide chains in water, endowing the modified film with a certain degree of water resistance. However, the composite film has a high water absorption rate but a relatively low water vapor transmission rate. It can absorb a significant amount of water and swell, but the tightly cross-linked network formed by the freeze–thaw treatment limits the further diffusion of water molecules across the membrane; the CMCS–Ag introduced during impregnation also fills some of the voids between polymer chains, further impeding water vapor transmission. This characteristic is significant for certain food packaging applications: the film can absorb trace amounts of moisture from the environment while also providing a certain degree of barrier against water vapor transmission.

### 3.12. Antibacterial Activity of CMC-CMCS-Ag Ternary Composite Films

**Figure 15:**
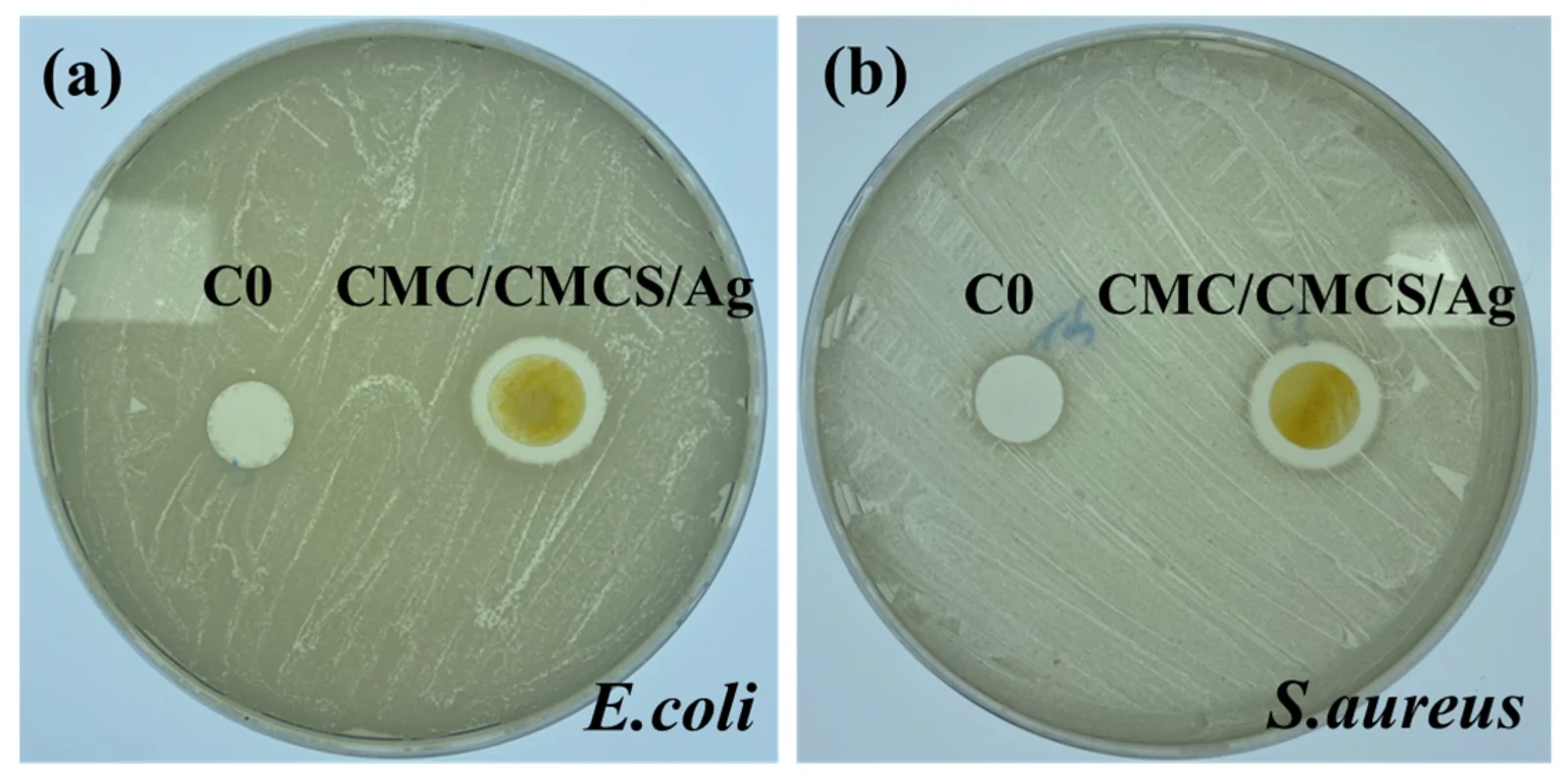
Antibacterial activity of C0 film and CMC-CMCS-Ag ternary composite film against: (**a**) *Escherichia coli*; (**b**) *Staphylococcus aureus*.

The antibacterial activities of the C0 film and the CMC-CMCS-Ag ternary composite film against *E. coli* and *S. aureus* are shown in Figure 15. The C0 film exhibited no antibacterial activity against either bacterium, as bacterial colonies remained densely grown around its circular sections. In contrast, the CMC-CMCS-Ag ternary composite film, due to the successful incorporation of AgNPs, produced clear inhibition zones around its sections. This is attributed to the ability of AgNPs to accumulate and adhere to bacterial surfaces. Gram-negative bacteria, with their thinner cell membranes, are more susceptible to membrane disruption, which increases membrane permeability and ultimately leads to cell death [52]. Furthermore, AgNPs can penetrate bacterial cells, disrupt intracellular structures, interfere with vital physiological activities, inhibit protein synthesis, suppress ribosome formation, bind to genetic material, and block transcription and translation, all of which contribute to bacterial death [53]. The inhibition zone diameters of the CMC-CMCS-Ag ternary composite film against *E. coli* and *S. aureus* were 14.1 mm and 14.7 mm, respectively, demonstrating a clear antibacterial effect against both strains. These results confirm that the prepared CMC-CMCS-Ag ternary composite film has been successfully endowed with antibacterial activity.

## 4. Conclusions

In this study, CMCS served as both the reducing and stabilizing agent for the in situ reduction of Ag^+^, yielding CMCS-Ag composites without any external chemical additives. The optimal synthesis conditions were identified as: 102 °C, 6 h, 10 mM AgNO_3_, and 0.05% CMCS. Under these conditions, the composites exhibited good stability and performance. The method is straightforward, requires no auxiliary reagents, and aligns with green chemistry principles. The CMCS-Ag composites were then impregnated into the H0 film to produce the CMC-CMCS-Ag ternary composite film. Mechanical tests showed that the ternary film achieved a tensile strength of 125.45 MPa, markedly higher than that of the pristine C0 film. Antibacterial assays revealed inhibition zones of 14.1 mm against *E. coli* and 14.7 mm against *S. aureus*, demonstrating clear antibacterial activity. Overall, this work offers a simple route to fabricating food packaging films with both enhanced mechanical strength and effective antibacterial performance, showing promise for applications in food preservation and safety.

## Figures and Tables

**Table 1:** Water absorption of CMC-CMCS-Ag ternary composite films after soaking for 0 h, 3 h, 6 h, 9 h and 12 h.

Time (h)	0	3	6	9	12
Weight (g)	0.0296	0.0557	0.0572	0.0577	0.0587
Water absorption (%)	-	88.18	93.24	94.93	98.31

**Table 2:** Water Vapor Permeability of the CMC-CMCS-Ag Ternary Composite Membrane.

Test Temperature	Humidity	Water Vapor Permeability
23.0 °C ± 0.5 °C	50 ± 2%RH	48.65 g/(m^2^·24 h)

## Data Availability

The data presented in this study are available on request from the corresponding author. Restrictions apply to the availability of these experimental data because the relevant raw material test data and sample preparation data are part of the ongoing follow-up research of the project, which cannot be publicly disclosed temporarily.
